# Emerging Systemic Therapies in Advanced Unresectable Biliary Tract Cancer: Review and Canadian Perspective †

**DOI:** 10.3390/curroncol29100555

**Published:** 2022-09-28

**Authors:** Vincent C. Tam, Ravi Ramjeesingh, Ronald Burkes, Eric M. Yoshida, Sarah Doucette, Howard J. Lim

**Affiliations:** 1 Division of Medical Oncology, Department of Oncology, University of Calgary, Calgary, AB T2N 4N2, Canada; 2 Division of Medical Oncology, Department of Medicine, Nova Scotia Health, Dalhousie University, Halifax, NS B3H 2Y9, Canada; 3 Division of Medical Oncology, Princess Margaret Cancer Centre, Mount Sinai Hospital, Toronto, ON M5G 1X5, Canada; 4 Division of Gastroenterology, Vancouver General Hospital, Vancouver, BC V5Z 1M9, Canada; 5 Medical Advisory Committee Chair, Canadian Liver Foundation, Markham, ON L3R 8T3, Canada; 6 IMPACT Medicom Inc., Toronto, ON M6S 3K2, Canada; 7 Division of Medical Oncology, BC Cancer, Vancouver, BC V5Z 4E6, Canada

**Keywords:** biliary tract cancer, rare disease, chemotherapy, immunotherapy, targeted therapy, drug access

## Abstract

Biliary tract cancer (BTC) is a group of rare and aggressive malignancies with a dismal prognosis. There is currently a significant lack in effective treatment options for BTC, with gemcitabine-cisplatin remaining the first-line standard of care treatment for over a decade. A wave of investigational therapies, including new chemotherapy combinations, immunotherapy, and biomarker-driven targeted therapy have demonstrated promising results in BTC, and there is hope for many of these therapies to be incorporated into the Canadian treatment landscape in the near future. This review discusses the emerging therapies under investigation for BTC and provides a perspective on how they may fit into Canadian practice, with a focus on the barriers to treatment access.

## 1. Background

Biliary tract cancer (BTC) is a group of aggressive malignancies arising from the gallbladder (gallbladder cancer, GBC), intrahepatic or extrahepatic bile ducts (cholangiocarcinoma, iCCA or eCCA), or ampulla of Vater (ampullary cancer) [1]. There is a paucity of literature describing the epidemiology of BTC, with the existing data suggesting that the incidence fluctuates over time and varies by region, sex, ethnicity, and subtype [2,3]. Globally, BTC has been reported to occur at rate between 1 and 4 cases per 100,000 people per year in most regions, with some regions exceeding an age-standardized annual incidence of 15 cases per 100,000 [2]. This qualifies BTC as a rare disease [4]. In Canada, one retrospective study of population-based cancer registries found an age standardized annual incidence rate of 2.1 cases per 100,000 people for gall bladder and extrahepatic cholangiocarcinoma (eCCA) [5]. A higher rate of gallbladder cancer has consistently been reported in women and a higher rate of eCCA has been reported in men [2,5]. As BTC is typically an aggressive disease, with few symptoms in the early stages, the majority are diagnosed in an advanced or metastatic stage where potentially curative surgery is not possible. The 5-year relative survival rate for patients with any-stage BTC is 9–10%, and is only 2% for patients with metastatic disease [6]. 

Currently, there is a lack of effective therapeutic options for patients with advanced BTC. For patients with resectable tumours, curative intent therapy with surgery followed by adjuvant chemotherapy is the standard of care, resulting in a median overall survival (OS) of over 4 years [7]. Radiotherapy may also be used as neoadjuvant or adjuvant therapy in conjunction with surgical resection, although the optimal methods for delivery of radiotherapy are unclear [8]. Nevertheless, only 20% of BTCs may be eligible for potentially curative resection [9]. Neoadjuvant chemoradiation followed by liver transplantation is an option for unresectable CCA without metastases, with a single-center study from the Mayo Clinic demonstrating a 4-year survival rate of 51% with this method [10]. However, the eligibility criteria to receive this therapy are strict. A similar chemoradiotherapy protocol used at Toronto General Hospital and Princess Margaret Cancer Centre showed a high rate of dropout and disease progression [11]. The current standard of care first-line treatment for patients with unresectable BTC is gemcitabine-cisplatin. This is based on the phase III ABC-02 trial which demonstrated significantly improved OS for gemcitabine-cisplatin compared to gemcitabine alone (up to 8 cycles; median OS 11.7 vs. 8.1 months; hazard ratio [HR] 0.64; 95% confidence interval [CI] 0.52–0.80) [12]. This standard of care has remained unchanged for over a decade as there has been no successful phase III trials to show superior survival benefit. 

Following progression on gemcitabine-cisplatin, many patients are not well enough to receive second-line therapy and there is a lack of effective treatment options for those fit to receive subsequent therapy [13]. While there are some promising targeted therapies for BTC, these are not funded in Canada and they could only benefit a small percentage of patients. In addition, patients who are elderly or have poor performance status are unable to tolerate chemotherapy, with their treatment limited to supportive care including decompression of the biliary tree through biliary stenting and ablation techniques [14]. This highlights the significant unmet need for more effective and tolerable treatment options in BTC, particularly in the first-line setting, and given the extremely poor prognosis for patients, it emphasizes the importance of patient-centered outcomes, such as quality of life and progression-free survival (PFS), in therapy selection.

**Figure 1:**
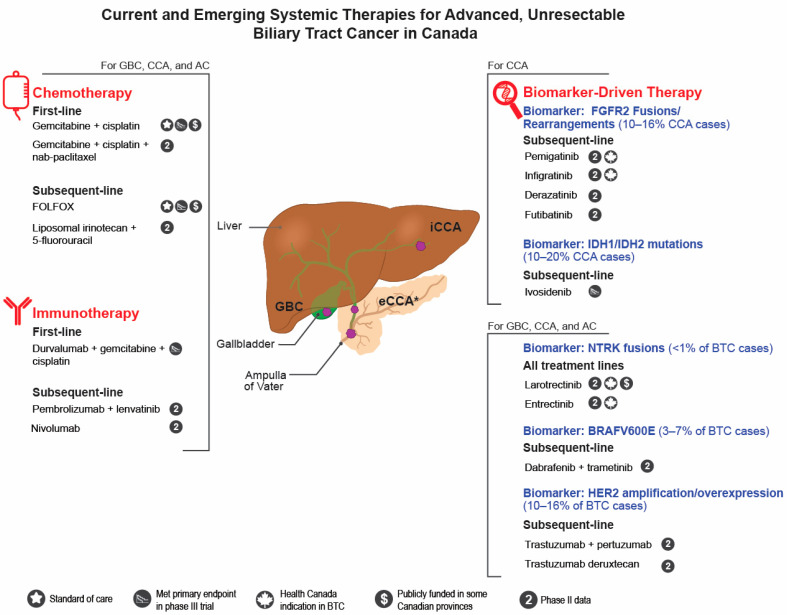
Current and emerging systemic therapies for advanced, unresectable biliary tract cancer in Canada. AC, Ampullary cancer; BTC, biliary tract cancer; CCA, cholangiocarcinoma; eCCA, extrahepatic cholangiocarcinoma; GBC, gallbladder cancer; iCCA, intrahepatic cholangiocarcinoma. * eCCA can be further subdivded into perihilar CCA which is proximal to the origin of the cystic duct and distal CCA which occurs between the cyctic duct and Ampulla of Vater.

Therapeutic development in BTC has flourished in the last few years, and after many unsuccessful clinical trials, there are finally novel treatments that can provide hope for patients with advanced, unresectable BTC. Several new regimens are expected to enter the treatment landscape in Canada within the next 5–10 years; however, significant barriers to accessing these therapies exist. Most therapies under investigation fit under the categories of new chemotherapy combinations, immunotherapy, and biomarker-driven targeted therapy (Figure 1). This review highlights promising therapies emerging for the treatment of advanced, unresectable BTC and provides a perspective on the barriers to accessing these treatments in Canada. 

## 2. Emerging Therapies in BTC

### 2.1. Chemotherapy

A number of chemotherapy combinations have been evaluated in clinical trials for the first-line treatment of advanced or metastatic BTC. None of these trials demonstrated survival outcomes beyond those achieved with gemcitabine-cisplatin in the ABC-02 trial; however, a recent phase II study evaluating albumin-bound paclitaxel added to gemcitabine-cisplatin in advanced BTC reported a median OS of 19.2 months, which compares favorably to the OS reported in the ABC-02 trial [15]. In addition, 20% of patients were down-staged to resectable disease. The phase III SWOG S1815 study evaluating gemcitabine-cisplatin in combination with albumin-bound paclitaxel or placebo is ongoing and will help clarify whether the chemotherapy triplet can achieve meaningful improvements in clinical outcomes over the chemotherapy doublet, without compromising quality of life or substantially increasing toxicity. 

Other triplet chemotherapy regimens have demonstrated less encouraging results when compared to gemcitabine-cisplatin. The PRODIGE38 study did not show an improved 6-month PFS rate for modified FOLFIRINOX compared to gemcitabine-cisplatin, which was the primary endpoint of the phase II portion of the study (44.6% vs. 47.3%) [16]. The median PFS and OS were also shorter in the modified FOLFIRINOX arm (median PFS: 6.2 vs. 7.4 months; median OS: 11.7 vs. 13.8 months).

Few randomized controlled trials have demonstrated efficacy of chemotherapy combinations in the second-line setting. The phase III ABC-06 study evaluating modified FOLFOX in patients with advanced BTC following first-line gemcitabine-cisplatin demonstrated a 15% improvement in 6- and 12-month survival rates compared to active symptom control alone [17]. The median OS was 6.2 months for modified FOLFOX versus 5.3 months for the control arm (HR 0.69; 95% CI 0.50–0.97; *p* = 0.031). This regimen has since become the most common second-line treatment option for patients with advanced BTC in Canada. Most recently, the phase II NIFTY trial, enrolling advanced BTC patients who progressed on first-line gemcitabine-cisplatin, reported a significantly longer median OS with liposomal irinotecan and 5-FU versus 5-FU alone (8.6 months vs. 5.5 months; HR 0.68; 95% CI 0.48–0.98; *p* = 0.035) [18].

### 2.2. Immunotherapy

Therapies targeting immune checkpoint pathways, including the programmed death-1 (PD-1)/programmed death-ligand 1(PD-L1) axis, have demonstrated activity in other cancers and have shown success in prolonging PFS and OS in both biomarker-selected and unselected populations [19]. The rationale for investigation of PD-1/PD-L1 targeted immunotherapy in BTC is based on the observation of PD-L1-expressing tumor cells and the presence of tumor-infiltrating CD8 T cells in the tumor microenvironment of BTC, both of which have been recognized as biomarkers of efficacy for PD-1/PD-L1 immunotherapy [20,21].

Single-agent immunotherapy with the anti-PD-1 agents pembrolizumab and nivolumab or the anti-PD-L1 agent durvalumab have demonstrated modest activity in phase II clinical trials of chemorefractory BTC, with overall response rates (ORRs) between 5 to 22% in unselected patients and 41% in patients with microsatellite instability/mismatch repair deficiency [22,23,24,25]. (Table 1) Dual checkpoint inhibition, targeting both the PD-1 and CTLA4 axis have also been explored, with similar outcomes to those achieved in single-agent trials [22,26] (Table 1).

There is evidence to suggest that chemotherapy may act synergistically with immunotherapy through several mechanisms of immunomodulation, providing a rationale to explore combination therapies in BTC [32]. Both nivolumab and durvalumab have been evaluated in phase II clinical trials in combination with gemcitabine-cisplatin for patients with unresectable or metastatic BTC. These trials reported median OS results of 10.6 months and 18.1 months, respectively [27,28]. 

The TOPAZ-1 study was subsequently conducted, which was a phase III, double-blind placebo-controlled trial randomizing patients with unresectable or metastatic BTC to receive gemcitabine-cisplatin (for up to eight cycles) plus durvalumab or gemcitabine-cisplatin (for up to eight cycles) plus placebo as first-line treatment [30]. The study met its primary endpoint showing a statistically significant improvement in OS for durvalumab plus gemcitabine-cisplatin versus placebo plus gemcitabine-cisplatin (median OS 12.9 vs. 11.3 months; HR, 0.76; 95% CI 0.64–0.91; *p* = 0.021) [31]. Addition of durvalumab to gemcitabine-cisplatin also prolonged PFS (median 7.2 vs. 5.7; HR 0.75; 95% CI 0.63–0.89; *p* = 0.001) and increased ORR (27% vs. 19%) [30]. Rates of grade 3/4 adverse events were not increased with the addition of durvalumab and 13% of patients experienced any grade immune related events (vs. 5% in the control arm). The triplet combination was well tolerated and quality of life was also maintained [33]. Another triplet chemo-immunotherapy regimen, pembrolizumab, gemcitabine, and cisplatin, is also being evaluated for patients with advanced BTC in the ongoing phase III KEYNOTE-966 trial [34]. 

Immune checkpoint inhibitors in combination with other novel agents and locoregional therapy are also being studied in BTC. Thus far, results have been reported for a phase II study evaluating the anti-VEGF agent lenvatinib in combination with pembrolizumab in advanced previously treated BTC. In this trial, pembrolizumab-lenvatinib achieved an ORR of 25%, median PFS of 4.9 months, and median OS of 11.0 months [29]. 

### 2.3. Biomarker-Driven Targeted Therapies

Several targeted agents have shown encouraging efficacy in patients with advanced BTC who harbor specific genomic alterations, although together, this represents a small fraction of patients with BTC and many of these patients may not be identified due to the limited access to biomarker testing for BTC in Canada. Recently, two agents targeting the Fibroblast Growth Factor Receptor (FGFR) family genes—pemigatinib and infigratinib—have been approved by Health Canada for previously treated, unresectable, or metastatic CCA with FGFR2 gene rearrangements. FGFR2 gene fusions occur in 10−16% of CCA cases, and almost exclusively occur in the intrahepatic subtype. The approval of pemigatinib and infigratinib were based on phase II studies where a median PFS of approximately 7 months was reached in patients with chemo-refractory cholangiocarcinoma with FGFR2 gene fusions [35,36]. (Table 2) A similar median PFS was achieved with erdafitinib in the LUC2001 trial (5.6 months), derazatinib in the FIDES-01 trial (8.0 months), and futibatinib in the FOENIX-CCA2 trial (8.9 months); all phase II studies of patients with advanced iCCA with FGFR2 fusions [37,38,39]. Notably, futibatinib has demonstrated some activity in patients previously treated with anti-FGFR agents, and both derazatinib and futibatinib have demonstrated similar activity in CCA with FGFR2 mutations as those with FGFR2 fusions/rearrangements [40,41]. Several ongoing phase III trials are evaluating first-line FGFR inhibitor therapy in patients with unresectable or metastatic CCA compared with gemcitabine-cisplatin. These include the PROOF (infigratinib, NCT03773302), FIGHT-302 (pemigatinib, NCT03656536), and FOENIX-CCA trials (futibatinib, NCT04093362).

Although rarely occurring in CCA (<1% of cases), tumors with gene fusions involving the neurotrophic tyrosine receptor kinase (NTRK) family proteins have been reported to respond to TRK inhibitors in a small number of patients evaluated in basket studies (2 of 3 patients with a partial response) [47,48]. Entrectinib and larotrectinib are currently approved by Health Canada for patients with unresectable locally advanced or metastatic solid tumors with NTRK gene fusions and no other satisfactory treatment options. 

Activating mutations in isocitrate dehydrogenase 1/2 (IDH1/2) occur in 10–20% of patients with CCA. The phase III ClarIDHy study randomized patients with previously treated advanced CCA harboring IDH1/2 mutations to treatment with the IDH1 inhibitor ivosidinib or placebo [42,49]. Ivosidinib led to a statistically significant improvement in PFS compared with placebo (2.7 vs. 1.4 months; HR, 0.37; 95% CI 0.25–0.54) (Table 2). No significant difference in OS was observed; however, this was likely caused by crossover of patients into the experimental arm. Several other agents targeting IDH1 and IDH2 are under investigation in BTC, including next-generation inhibitors aimed at escaping resistance mechanisms acquired after ivosidenib treatment [50].

Mutations in the BRAF gene have been reported in 3–7% of BTC cases, and are also enriched in those with iCCA. In a basket study exploring the activity of the BRAF and MEK inhibitors dabrafenib and trametinib in rare tumours harboring the BRAFV600E mutation, the cohort of 43 patients with BTC achieved an ORR of 51% and median OS of 14 months [43]. Encouraging activity for this combination was also reported in the NCI-MATCH trial subprotocol H, with 3 of 4 patients with BRAFV600E-mutated BTC achieving a partial response [51].

HER2 amplification or overexpression occurs in 10–16% of gallbladder carcinomas and 5–11% of eCCA [52]. Given the success of anti-HER2 therapies in other solid tumors expressing HER2, several phase I/II studies have evaluated these therapies in BTC [50]. Clinical trials of pertuzumab-trastuzumab and trastuzumab deruxtecan in patients with previously treated metastatic BTC have reported ORRs of 22% and 36%, respectively, and median OS results of 10.9 months and 7.1 months [44,45] (Table 2). Trastuzumab is now being tested in combination with gemcitabine-cisplatin in the phase II BILHER study (NCT03613168). In a study of patients with metastatic treatment-refractory BTC and HER2 mutations, the irreversible HER2 kinase neratinib achieved an ORR of 16% and median OS of 5.4 months [46].

Increasingly sophisticated genomic analyses in BTC are expected to reveal insights on additional biomarkers of treatment response and new therapeutic targets. For example, a multi-omics analysis in iCCA tumor samples identified an IDH mutant-enriched subtype that correlated with hypermethylation and decreased expression of ARID1A, suggesting that inhibition of chromatin modifiers such as EZH2 may be effective in this subtype [53]. Other studies have reported a high frequency of other actionable biomarkers in BTC including PTEN, CDKN2A, and KRAS which warrant further investigation in clinical trials [54,55]. Outside of microsatellite instability, biomarkers that can predict response to immune checkpoint inhibitors have been unsuccessful thus far, likely due to the heterogeneity of immune cells in the tumor microenvironment of BTC. Single-cell RNA sequencing techniques can characterize complex immune cell populations in the tumor microenvironment and have shown promise in identifying biomarkers for immunotherapy response [56]. Together, this emphasizes the continued role precision medicine will play in improving treatment response in BTC.

## 3. Canadian Perspective on Access to Therapies in BTC

With several recent BTC trials reporting encouraging results, the question on many Canadian oncologists’ minds is which therapies will be made available to our patients and funded by the Canadian provinces. Following Health Canada approval, the decision to provide Canadians with access to drugs through public reimbursement programs is made at the provincial level. Funding decisions are largely aided by recommendations from Health Technology Assessment (HTA) bodies, including the Canadian Agency for Drugs and Technologies in Health (CADTH) and Quebec’s Institut National d’Excellence en Santé et en Services Sociaux (INESSS). These HTA bodies consider cost-effectiveness, patient-based values, and adoption feasibility in addition to clinical benefit, when appraising drugs for reimbursement recommendations [57]. 

As BTC is a rare, aggressive disease with extremely poor prognosis and limited treatment advances, access to new therapies can be hindered if HTA assessments (specifically clinical value and cost-effectiveness) are measured against the same standards as all oncology drugs. For example, although phase III randomized controlled trials are the gold standard for determining clinical benefit, this trial design may be difficult to achieve in BTC, particularly in the second-line setting, as trial enrollment is challenged by a small overall population of eligible patients. Evaluation of targeted agents that require the selection of even smaller genetically defined subpopulations adds to enrollment difficulty. This challenge is illustrated in the negative recommendation issued by CADTH for the reimbursement of pemigatinib in patients with previously treated CCA with FGFR2 fusions [58]. This recommendation was based on the uncertainty that pemigatinib filled the patient-identified needs of improved tumor response, delayed disease progression, and improved quality of life, given the single-arm, open-label design of the phase II FIGHT-202 trial; despite the acknowledgment that a phase III randomized controlled trial would be unfeasible in this setting.

In contrast to the negative recommendation for pemigatinib, the TRK inhibitor larotrectinib has been issued a conditional positive recommendation for funding by CADTH, for any patient with an advanced solid tumor harboring NTRK fusions who have no other effective treatment options [59]. This followed an initial negative recommendation based on uncertainty in the clinical benefit of larotrectinib given the heterogeneity of patients enrolled in the three single-arm phase I/II trials, among other reasons. The current positive recommendation was based on updated pooled analyses of the aforementioned trials, as well as supportive real-world data. This analysis demonstrated ORRs of 73% in the overall population and median PFS of approximately 33 months. Although, with only two patients with CCA enrolling, it is unclear whether the efficacy results for larotrectinib can be generalizable to patients with BTC. Furthermore, patients with NTRK fusions represent <1% of the population across many cancers, including BTC, and variable access to testing across provinces may impede access to this therapy [60,61].

Another challenge with assessing new therapies in BTC is deciding what constitutes a clinically meaningful benefit. Both the American Society of Clinical Oncology (ASCO) and the European Society for Medical Oncology (ESMO) have published consensus documents proposing how a clinically meaningful benefit may be measured across different tumor types and scenarios. In ASCO’s publication, working groups for colon, pancreatic, lung, and breast cancer all selected median OS as the primary end point of interest. In general, they deemed an HR between 0.6 to 0.8 and a median OS improvement between 2.5 to 6 months over standard therapy as a clinically meaningful outcome [62]. However, the group acknowledged that the definition of a clinically meaningful benefit is nuanced and may be influenced by other factors including clinical context, effectiveness, toxicity, and patient goals and preferences. Thus, consensus values should not be used to set standards for regulatory approval or funding. It is unclear whether these benchmarks are applicable to BTC, where the inability to design trials with a large sample size, as is common in lung, colon, and breast trials, may prevent such hazard ratios from being achieved.

Response rate, duration of response, and PFS, although not validated as surrogate markers for OS, are also clinically meaningful in BTC. A large, durable response can downstage patients, as was reported in 20% of patients in the phase II trial of albumin-bound paclitaxel plus gemcitabine-cisplatin [15]. This may allow them to receive potentially curative surgery. Retrospective studies have observed similar survival outcomes after surgery in patients with initially unresectable localized iCCA that were down-staged following chemotherapy compared with initially resectable patients [63,64]. In addition, given the small size of the biliary tract, even minimal tumor growth can lead to disease symptoms causing significant deterioration of quality of life and necessitate stent placements or changes. These are associated with complications requiring hospitalization, including bleeding, perforation, cholangitis, and infection [65]. Consideration of these outcomes in future value assessments may better reflect the unique circumstances and needs of patients with BTC.

Quality of life is also of great importance to patients with BTC and must be considered in the value assessment of a therapy [66]. The Magnitude of Clinical Benefit Scale created by an ESMO working group (ESMO-MCBS) incorporates quality of life measures by increasing the clinical benefit score of a drug if quality of life and/or major toxicity is improved, such that drugs demonstrating a smaller magnitude of benefit for efficacy may still be categorized as substantially beneficial if these criteria are met [67]. 

The prospect of patients with BTC gaining access to new treatments is exciting. However, the only regimen currently being reviewed for Health Canada approval is durvalumab in combination with gemcitabine-cisplatin for first-line treatment of BTC. This is based on results from the phase III TOPAZ-1 trial, which is the first phase III randomized, double-blind, placebo-controlled trial in over a decade to demonstrate a statistically significant improvement in OS, without increasing toxicity or reducing quality of life, compared to gemcitabine-cisplatin alone [30,33]. However, the TOPAZ-1 trial has potential limitations that may impact HTA assessment and use in Canada, particularly if it is assessed against the same standards traditionally used in other cancers. Although an improvement in median OS of 1.6 months and a HR of 0.76 may be a meaningful benefit to some patients and caregivers, it is uncertain whether this outcome alone will meet the value thresholds set by CADTH who have historically issued few positive recommendations for reimbursement where the improvement of median OS was below 2 months [57].

As demonstrated in other clinical trials, median OS alone does not appropriately capture the extended right-sided tail commonly observed in the survival curves for chemoimmunotherapy regimens, which represents a portion of patients with long-term survival [68,69,70]. In such cases, analysis of survival rate at a set milestone is suggested to better capture the incremental effect of the experimental treatment [71]. At the current follow-up of 23 months, the survival curves from TOPAZ-1 show a potential right-tail plateau forming, corresponding to an improved 24-month OS rate for gemcitabine-cisplatin plus durvalumab over gemcitabine-cisplatin plus placebo (23.6% vs. 11.5%) [31]. With an improvement in 2-year OS rate exceeding 10%, durvalumab in combination with gemcitabine-cisplatin has earned a score of 4 points on the ESMO-MCBS, representing a substantial clinical benefit [72,73].

Another potential limitation of the TOPAZ-1 trial comes from the dosing schedule of gemcitabine-cisplatin. In TOPAZ-1, gemcitabine-cisplatin was stopped after eight cycles of therapy in both arms, similar to the dosing regimen used in the ABC-02 trial. However, anecdotally, many Canadian oncologists will give gemcitabine-cisplatin until progression or dose-limiting toxicity (such as neuropathy related to cisplatin), or treatment is continued with gemcitabine monotherapy after cycle 8. This may complicate the implementation of durvalumab for the first-line treatment of BTC specifically in Canada. The practice of giving gemcitabine-cisplatin until progression or toxicity is largely based on studies from other tumor types such as breast cancer, which demonstrate improved survival with continued palliative chemotherapy [74]. One retrospective observational study from Canada suggests that some patients may benefit from continued chemotherapy [75], while other studies have not observed a clear benefit [76]. The ongoing KEYNOTE-966 trial allows gemcitabine to be given with pembrolizumab or placebo beyond eight cycles (until progressive disease or unacceptable toxicity), which may better reflect the practice of some Canadian oncologists [34]. 

## 4. Conclusions

The current landscape of systemic treatments for advanced, unresectable BTC is extremely limited in Canada, consisting mainly of chemotherapy options including standard of care first-line treatment with gemcitabine-cisplatin. Although biomarker-driven agents targeted against NTRK (larotrectinib, entrectinib) and FGFR (pemigatinib, and infigratinib) are approved by Health Canada for second-line therapy, there are several barriers to accessing these agents for patients with advanced BTC. These include lack of provincial funding and access to timely biomarker testing. In addition, the pool of patients who harbor FGFR2 or NTRK fusions and who are fit to receive second-line therapy and beyond represent less than 5% of the BTC population. Therefore, there is still a high unmet need for more effective therapies for all patients with BTC, particularly in the first-line setting.

The wave of chemo-immunotherapy and biomarker-driven treatments showing activity in clinical trials for advanced BTC is reminiscent of the therapeutic evolution in non-small cell lung cancer that began 10 years ago, which provided much needed new treatment options for patients with a very poor prognosis. However, in contrast to the rarity of BTC, the high frequency of lung cancer in Canada and throughout the world allowed for large, randomized phase III trials to be done which demonstrated a clear clinical benefit. Given these large trials are challenging to conduct in BTC, different considerations for value assessment beyond median OS are needed. Progression-free survival, response rate, duration of response, and quality of life are particularly important outcomes in BTC. Landmark OS analyses that can capture whether a portion of patients can achieve long-term survival from an experimental treatment are also valuable. To better understand how new therapies might provide value to patients, clinical trials and real-world studies should aim to capture outcomes such as biliary stenting, hospitalizations, and down-staging, and explore whether these correlate with patient quality of life. Together, this may help to shape the definition of a clinically meaningful benefit for patients with BTC and improve access to therapies that meet the needs of patients.

## Figures and Tables

**Table 1:** Results reported from phase II/III clinical trials investigating immune checkpoint inhibitors in advanced BTC

Trial Name/Phase	Treatment Arms	Population	ORR	Median PFS	Median OS
NCT02829918 [24] Phase 2	Nivolumab	Advanced BTC Second or third line N = 54	22%	3.7 months	14.2 months
KEYNOTE-158 [23,25] (NCT02628067) Phase II	Pembrolizumab	Advanced MSS BTC Second line and beyond N = 104 Advanced MSI-H cholangiocarcinoma N = 22	MSS: 5.8% MSI-H: 40.9%	2 months 4.2 months	9.1 months 24.3 months
CA209-538 [26] (NCT02923934) Phase II	Nivolumab + ipilimumab	Advanced BTC First-line and beyond N = 39	23%	2.9 months	5.7 months
BilT-01 [27] (NCT03101566) Phase II	Arm A: Nivolumab + Gem-Cis Arm B: Nivolumab + ipilimumab	Advanced BTC First-line N = 71	NR	Arm A: 7.4 months Arm B: 4.1 months	Arm A: 10.6 months Arm B: 8.3 months
NCT03046862 [28] Phase II	Arm A: Gem-Cis → Gem-Cis + durvalumab Arm B: Gem-Cis + durvalumab Arm C: Gem-Cis + durvalumab + tremelimumab	Advanced BTC First-line N = 121	Arm A: 50.0% Arm B: 73.4% Arm C: 73.3%	Arm A: 13.0 months Arm B: 11.0 months Arm C: 11.9 months	Arm A: 15.0 months Arm B: 18.1 months Arm C: 20.7 months
NCT03895970 [29] Phase II	Pembrolizumab, Lenvatinib	Advanced BTC Second line and beyond N = 32	25%	4.9 months	11.0 months
TOPAZ-1 [30,31] Phase III	Durvalumab + Gemc-Cis vs. Placebo + Gem-Cis	Advanced, unresectable BTC First-line N = 341	ORR_(durva vs. placebo)_ 26.7% vs. 18.7% OR_(durva vs. placebo)_ 1.60 (95% CI 1.11−2.31)	mPFS_(durva vs. placebo):_ 7.2 vs. 5.7 months HR_(durva vs. placebo)_ 0.75 (95% CI 0.63−0.89) *p* = 0.001	mOS_(durva vs. placebo)_ 12.9 vs. 11.3 months HR_(durva vs. placebo)_ 0.76 (95% CI 0.64−0.91)

BTC, biliary tract cancer; CI, confidence interval; durva, durvalumab; Gem-Cis, gemcitabine-cisplatin; HR, hazard ratio; m, median; MSI-H, microsatellite instability high; MSS, microsatellite stable; NR, not reported; OR, odds ratio; ORR, overall survival; OS, overall survival; PFS, progression-free survival.

**Table 2:** Results reported from phase II/III clinical trials investigating biomarker-driven targeted therapy in >20 patients with advanced BTC.

Trial Name/Phase	Treatment Arms	Target/ Biomarker	Population	ORR	Median PFS	Median OS
FIGHT-202 [35] (NCT02924376) Phase II	Pemigatinib	FGFR1-3 FGFR2 fusions	Chemotherapy refractory advanced iCCA N = 107	36%	6.9 months	21.1 months
NCT02150967 [36] Phase II	Infigratinib	FGFR1-4 FGFR fusions	Chemotherapy refractory advanced iCCA N = 71	31%	6.8 months	12.5 months
FIDES-01 [37] (NCT03230318) Phase II	Derazatinib	FGFR1-3 FGFR2 fusions	Chemotherapy refractory advanced iCCA N = 103	21.4%	8.0 months	15.9 months
FOENIX-CCA2 [38] (NCT02052778) Phase II	Futibatinib	FGFR1-4 FGFR fusions	Chemotherapy refractory advanced iCCA N = 103	41.7%	8.9 months	20.0 months
ClarIDHy [42] (NCT02989857) Phase III	Ivosidenib vs. Placebo	IDH1	Advanced/metastatic CCA Second line N = 187	2%	mPFS_(ivo vs. plb)_ 2.7 vs. 1.4 months HR_(ivo vs. plb)_ 0.37 (95% CI 0.25−0.54) *p* = 0.001	mOS_(ivo vs. plb)_ 10.3 vs. 7.5 months HR_(ivo vs. plb)_ 0.79 (95% CI 0.56−1.12) *p* = 0.09
ROAR [43] (NCT02034110) Phase II	Dabrafenib + trametinib	BRAF + MEK BRAF V600E	Advanced/metastatic CCA Second line N = 43	51%	9 months	14 months
MyPathway [44] (NCT02091141) Phase II	Pertuzumab + trastuzumab	HER2 HER2 amplification/overexpression	Previously treated metastatic BTC N = 39	23%	4 months	10.9 months
HERB [45] (JMA-IIA00423) Phase II	Trastuzumab deruxtecan	HER2 HER2 amplification/overexpression	Previously treated metastatic BTC N = 22	36.4%	4.4 months	7.1 months
SUMMIT [46] (NCT01953926) Phase II	Neratinib	HER2 HER2 somatic mutation	Previously treated metastatic BTC N = 25	16%	2.8 months	5.4 months

BTC, biliary tract cancer; CI, confidence interval; CCA, cholangiocarcinoma; HR, hazard ratio; iCCA, intrahepatic cholangiocarcinoma; ivo, ivosidenib; m, median; NR, not reported; ORR, overall survival; OS, overall survival; PFS, progression-free survival; plb, placebo.

## Data Availability

Not applicable.
